# Giant Cell Reparative Granuloma of the Orbit: Clinicopathological Characteristics and Treatment

**DOI:** 10.1155/2021/4917968

**Published:** 2021-05-27

**Authors:** Álvaro Bengoa-González, Enrique Mencía-Gutiérrez, Beatriz Alonso-Martín, Bianca-Maria Laslău, Elena Salvador, Ana-Belén Enguita-Valls, María-Dolores Lago-Llinás

**Affiliations:** ^1^Ophthalmology Department, 12 de Octubre Hospital, Complutense University, 28041 Madrid, Spain; ^2^Radiology Department, 12 de Octubre Hospital, Complutense University, 28041 Madrid, Spain; ^3^Pathology Department, 12 de Octubre Hospital, Complutense University, 28041 Madrid, Spain

## Abstract

Giant cell reparative granuloma (GCRG) is a rare fibroosseous lesion uncommonly seen in the orbital area. Although benign, it is known to be recurrent and locally destructive. We report two cases of GCRG of the orbit. In both cases, computed tomography revealed a heterogeneously growing well-defined mass, arising from the roof of the orbit, affecting the cortex, and invading the orbit. In the first case, the mass extended into the anterior cranial fossa. Magnetic resonance imaging with gadolinium showed, in both cases, a cystic character of the lesion with fluid levels. The surgical treatment was performed via an upper crease incision. An ultrasonic aspirator system was used to remove the tumor tissue and its extension into cranial fossa. Careful histopathologic analysis established the diagnosis of GCRG. Symptoms resolved completely with no evidence of recurrence after a follow-up of 18 and 14 months, respectively. We present the clinicopathological and radiological findings, and we describe the surgical approach. As a rare entity, GCRG of the orbit should be considered in differential diagnosis of fibroosseous orbital masses. Complete surgical excision carries a low risk of recurrence.

## 1. Introduction

Giant cell reparative granuloma (GCRG) is an uncommon osteolytic lesion that typically develops in the jawbone [1, 2], although it has also been described in other locations such as maxilla, sphenoid, ethmoid, and temporal bones and it also can be found occasionally in the small bones of the hands and feet [3].

It was Jaffe [4], who suggested this term in 1953 to describe lesions of the mandible and maxilla that were thought to occur as a nonneoplastic reaction secondary to an intraosseous hemorrhage. GCRG of the orbit is a very rare occurrence and was first reported by Sood et al. in 1967 [3–6].

It is included within a group of orbital fibroosseous lesions, among which are the osteoma, ossifying fibroma, fibrous dysplasia, osteoblastoma, osteoclastoma, brown tumor of hyperparathyroidism, and the aneurysmal bone cyst [1], with overlapping clinical and histopathologic features [7].

We have found 10 cases of GCRG with orbital involvement published in the medical literature [5], and only two of them had intracranial invasion [8, 9].

We present two cases of orbital GCRG, one of them with extension into the anterior cranial fossa. We describe the clinical, histological, and radiological characteristics and the surgical approach to removing them using an ultrasonic surgical aspirator device.

## 2. Case Presentation

### 2.1. Case 1

**Figure 1:**
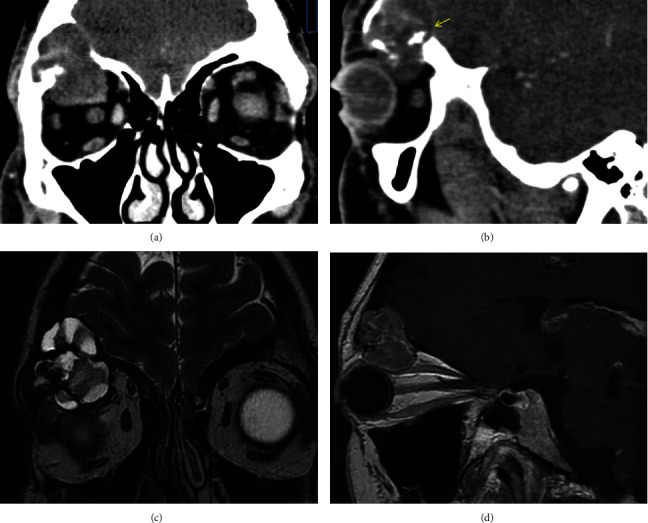
(a, b) Contrast-enhanced coronal and sagittal CT images, demonstrating an expansive lesion arising from the roof of the right orbit with a heterogeneous enhancement and soft tissue attenuation. Inside view of the lesion reveals several small foci of mineralization (arrow). The lesion shows the osseous expansive changes, with thinning of superior and inferior wall of the roof which leads to an invasion over the frontal sinus and into the orbit. (c) Coronal T2-weighted MRI shows a well-defined lesion with a low-signal-intensity margin representing either osseous sclerosis or a pseudocapsule. The lesion shows a multilobulated lytic pattern which reveals markedly increased signal intensity, reflecting the expansive cystic component, and low signal intensity in the small solid regions. (d) Sagittal postcontrast T1-weighted MR image shows a well-encapsulated mass with homogenous contrast enhancement.

**Figure 2:**
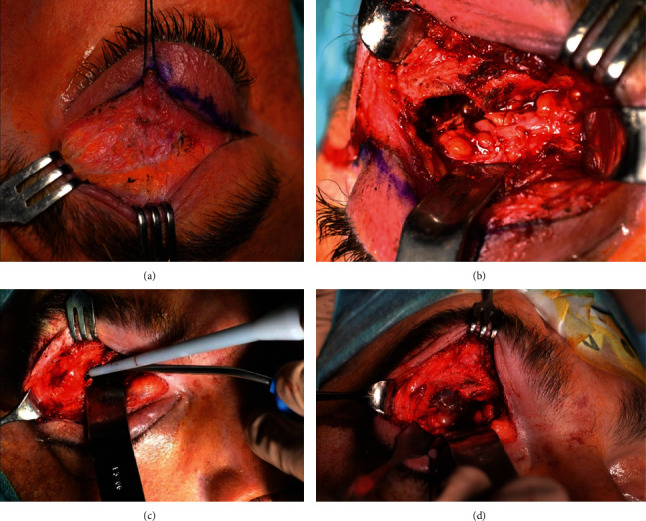
(a) Surgical approach through an upper eyelid crease incision to access the orbital roof. Intraoperative photograph showing bone destruction in the roof of the orbit and the presence of a red-yellowish mass, with evidence of dark coagulated blood and fragments of bone within the soft tissue. The tumor extends into the anterior cranial fossa. (b) The handpiece of SONOPET® ultrasonic aspirator used for aspiration and emulsification of the tumor tissue in the superior orbit and its extension into cranial cavity. (c) Resection of abnormal tissue from the upper orbit and its extension into anterior fossa.

A 37-year-old male presented with proptosis, fullness of the right upper eyelid, and inferior displacement of the right eye that had progressed gradually over several months. There was no history of pain, loss of visual acuity (VA), or diplopia. Ocular motility examination revealed a slight limitation of motion in supraduction. The patient reported no previous episodes of inflammation, sinus infection, or trauma. Fundus examination was normal. General physical examination and routine blood tests were within normal limits. Contrast-enhanced coronal and sagittal computed tomography (CT) images showed a lytic lesion arising from the roof of the right orbit, with heterogeneous captation, relatively hypocaptant with hypodense areas, and small foci of mineralization within the lesion. The mass had a thinning effect on the superior wall of the roof, without breaking it, and extended over the anterior cranial fossa. It also disrupted the inferior wall of the roof and invaded the orbit, displacing the superior rectus muscle and the globe inferiorly (Figures 1(a) and 1(b)). Magnetic resonance imaging (MRI) revealed a well-defined, heterogeneously enhancing mass, measuring 30 mm × 19 mm × 27 mm, hyperintense on T1-weighted images, and with lobulated, “pseudonodular” appearance on T2-weighted images, with markedly increased signal intensity, reflecting the expansible cystic component (Figures 1(c) and 1(d)). An orbitotomy was performed via an upper eyelid crease incision, and a red-yellowish friable mass, with evidence of dark coagulated blood, and fragments of bone tissue within the soft tissue was observed (Figure 2(a)). A tissue sample was taken for histopathological study, and then, an ultrasonic aspirator system (SONOPET®, Stryker, Kalamazoo, MI, USA) was used to remove the tumor. The handpiece of this device, with a soft tissue straight tip attached, was used to aspirate and separate the tumor from the surrounding heathy tissue, preventing damage to it. The handpiece is lightweight and ergonomic, allowing it to be inserted into narrow spaces with poor visibility, thus making possible the approach to the cranial fossa through the orbital roof (Figure 2(b)). Moreover, with the Spetzler micro claw tip, the lateral edge of the orbital roof was sculpted to gain visibility and access to the cranial fossa. Once the tumor tissue was removed, we performed a curettage, removing the capsule and the tissue attached to the cortical surface of bone (Figures 2(c) and 2(d)). During these surgical maneuvers, a leak of cerebral spinal fluid, originating in the innermost part of the cavity, was noted and sealed with human fibrinogen/human thrombin, TachoSil®, Talceda Austria GmbH, Linz, Austria, efficiently. Oral treatment with antibiotics was prescribed for 1 week. Symptoms resolved and there was no evidence of recurrence after 18 months of follow-up.

### 2.2. Case 2

**Figure 3:**
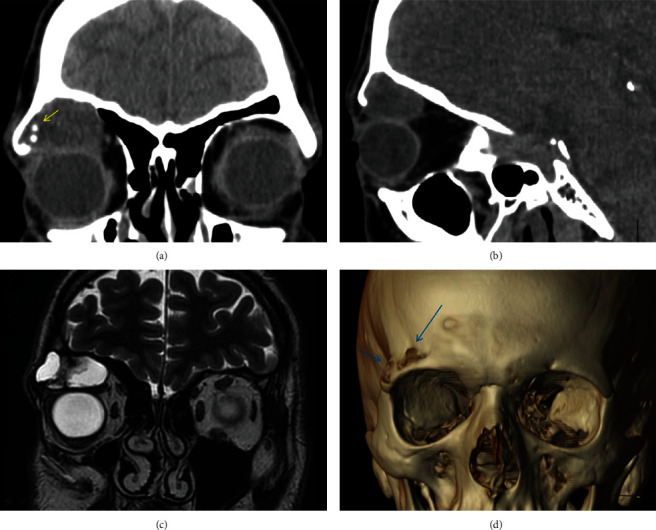
(a, b) Coronal and sagittal CT images, demonstrating an expansive lesion arising from the roof of the right orbit with soft tissue attenuation. Small foci of mineralization (arrow) into the lesion are presented. The lesion protrudes into the orbit. (c) Coronal T2-weighted MR image shows a well-defined lesion arising from the bone with an extraosseous component. The majority of the tumor has high signal intensity on the T2-weighted image, with low signal areas which represent the solid component of the tumor. (d) Volume-rendered 3D-CT reconstruction images show lytic areas with bone destruction in the roof of the orbit.

**Figure 4:**
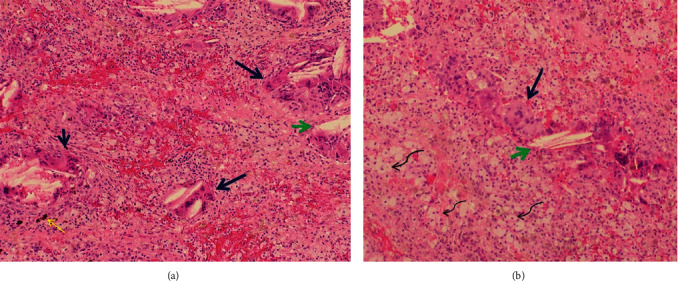
(a, b) Histopathology (hematoxylin and eosin stain (40x) showing multinucleated foreign body giant cells (cholesterol crystals) with xanthomized cells, presence of hemosiderin deposition, and lymphocytic inflammatory infiltrate. Multinucleated giant cells (thick blue arrows), cholesterol crystals (short green arrows), xanthomized cells (wavy black arrows), and hemosiderin deposits (fine yellow arrow).

A 39-year-old male was referred to our oculoplastic service complaining of gradually progressive proptosis, and right upper eyelid swelling. The VA and ocular motility examination were normal. He had previous episodes of sinusitis and endoscopic surgical repair of the right frontal sinus. Orbital CT revealed a cystic mass measuring 30 mm × 15 mm × 20 mm that eroded the orbital roof, with thinning and pushing the cortex, but without invading the cranial cavity (Figures 3(a)–3(c)). MRI showed a well-defined mass, adjacent to the frontal sinus, of heterogeneous tissue. The majority of the tumor had high signal intensity on the T2-weighted image, with low signal areas which represented the solid component of the tumor (Figure 3(c)). Surgical treatment was performed via an upper eyelid crease incision. Gross examination of the specimen showed a soft, friable, red-bluish hemorrhagic tissue with small fragments of bone tissue within. A tissue sample was taken, followed by surgical excision using the ultrasonic aspirator system. The curettage was carried out eliminating the attachment to the orbital walls without incident. Symptoms resolved completely without recurrence after 14 months of follow-up. The histopathological study of both cases showed similar features. It consisted of a cellularity with a fibrohistiocytic appearance, without atypia, with xanthomized cells as well as pigmented areas with hemosiderin deposits. In addition, there was a foreign body reaction and cholesterol crystals. Osteoid tissue was not identified. There were no areas of malignancy (Figures 4(a) and 4(b)). These findings led to the diagnosis of reparative giant cell granuloma in both cases. The biochemical study of the blood, including electrolytes, was within normal values.

## 3. Discussion

GCRG of the orbit is a benign fibrous lesion, although it can expand aggressively and can be locally destructive [1, 3], which happened in the cases which we present.

The etiology of this tumor remained unclear [8] but may be related to a reparative granulomatous response to an intraosseous hemorrhage [1, 5]. It is thought that chronic inflammation and trauma to the paranasal sinuses could produce intraosseous hemorrhage that initiates the reparative process [10], which might be the etiology of the second case we presented, although a history of trauma or chronic sinusitis has never been consistently demonstrated in all patients with GCRG [3, 10].

GCRG occurs more frequently in women and in the first two decades of life [11]. Almost all previously published orbital cases have been reported in patients under 40 years of age, like those presented in this study, but Pherwani et al. reported [3] a GCRG in an 85-year-old patient.

The clinical signs that our patients mainly manifested were proptosis, upper eyelid fullness, periorbital inflammation, lower displacement of the globe, and impaired extraocular movements; these signs and symptoms are in line with those described in the medical literature [1, 8, 9, 12]. Although not in our patients, other symptoms such as headache [13], decreased VA [9], orbital deformity [6, 9], and diplopia [14] have also been reported.

Most of the published cases of GCRG of the orbit affected the roof and/or the lateral orbital wall, as in our cases, but it can also affect the inferior, central, and medial portion of the orbit (Table 1) [1, 3, 5, 6, 8, 9, 12–15].

Treatment of GCRG is surgical excision, which is usually done with total resection or local curettage [1, 16], although complete surgical resection could not be done safely in some cases, due to tumor location and proximity to delicate structures [17].

Imaging findings of GCRG are not specific, thus making it very difficult to distinguish from other osteolytic bone lesions [18]. In both of our cases, the CT scan showed a heterogeneously growing [1] well-defined mass arising from the roof of the orbit, affecting the cortex and invading the orbital space. In the first case, cortical thinning and bulging were observed, as noted by Sebag et al. [8], who describe a cavity that invades the cranial fossa. Although in the first case the CT scan did not show a cortical tear, and a connection to the extradural space was ruled out, when detaching the mass tissue from the thin layer of the cortical bone, we observed a CSF leak which was sealed with TachoSil® (human fibrinogen/human trombin) efficiently.

Given the location and extent of the mass, an upper eyelid crease approach was used to access the superior orbital space, the orbital roof, and cranial fossa extension. Intraoperatively, bone destruction of the orbital walls was noted, and detached bone fragments were observed among soft tissues.

On gross inspection, the tumor may appear as a friable red-bluish mass [1], although it may also be cystic, bluish, fluctuating, and multiloculated, containing dark coagulated blood [1, 5, 8]. Histologically, GCGR is characterized by the presence of multinucleated giant cells, with or without new bone formation, intermingled with inflammatory cells, and focal areas of hemorrhage [18].

The GCGR of the orbit is very similar to the brown tumor of hyperparathyroidism, although in the latter the serum levels of calcium are usually high and those of phosphorus are low [1, 3]; its levels were normal in our cases.

It is important to distinguish GCRG from other tumors, such as osteoclastoma, also known as giant cell tumor, for management and prognosis. Osteoclastoma is a giant cell tumor within the bone [1] that mostly involves the epiphysis of long bones [9, 10]. It usually appears in the third to fourth decade of life, being very rare in those under 18 years of age. This tumor is composed of multinucleated giant cells distributed diffusely and evenly, whereas in GCGR the multinucleated giant cells are smaller, irregular, and clustered around hemorrhagic foci [1, 5]. New bone foci can also be observed in GCGR, but not in osteoclastoma [9, 10, 19].

Eosinophilic granuloma is the bone variant of histiocytosis X or Langerhans cell histiocytosis and generally occurs in the first decade of life [20]. In the cases presented, the histological image and the immunohistochemical profile ruled out that these were cases of an eosinophilic granuloma nor were cystic images observed that would have led to the diagnosis of an aneurysmal bone cyst; in addition, this bone cyst more frequently is located in vertebrae and metaphysis of long bones [12].

In both cases, an ultrasonic aspirator system was used to remove the tumor. The SONOPET® ultrasonic aspirator is a handheld surgical tool that allows access to small operative fields, such as the orbit [21]. The design of the handpiece and its weight allowed us to maneuver it for the extraction of tumors that are difficult to reach, as was the situation in the cases we present. In our first case, the mass expanded into the cranial fossa and was removed using an orbital approach, thus avoiding a craniotomy, as performed by other authors [8, 22, 23].

This ultrasonic system allowed us to sculpt the bone precisely, when an improvement of visibility was needed, as in the first case presented, and to remove the abnormal tissue, regardless of its consistency, with no traction nor sharp excision. It enables work near areas such as the dura mater without damaging the adjacent tissues.

We think, as do other authors [24, 25], that this device is particularly helpful in removing and sculpting, in a simple, secure, and precise way, infiltrative orbital masses with difficult anatomical access, as was the situation in our cases [26].

The recurrence rate of these tumors can be between 10 and 15% of the cases that have been incompletely removed, as has been seen in other published cases [6, 9]. The mean interval from the initial treatment to recurrence has been described around 19 months [27]; therefore, our patients need continuous follow-up on a regular basis due to the relatively short follow-up time of our two cases. Although spontaneous resolution has been documented [10], recurrences might be treated with reoperation or radiotherapy, options that can help in achieving complete remission [4, 12]. However, radiotherapy is generally not advisable due to the risk of carcinomatous or sarcomatous transformation and should be reserved for patients who are not surgical candidates [9].

## 4. Conclusions

In conclusion, the orbital GCGR, although rare, should be included in the differential diagnosis of fibroosseous lesions or other orbital masses. Although it is more frequent in young or middle-aged patients it must be considered in older patients too. The surgical resection of this lesion is usually possible and can be curative, relieving ocular symptoms.

## Figures and Tables

**Table 1:** Giant cell reparative granuloma cases involving the orbit.

Authors	Number cases	Side, location in the orbit
Bengoa-González A et al., current study	2	Right, superior, one extending anterior cranial fossa
2013, Chawla et al. [5]	1	Right, lateral
2005, Pherwani et al. [3]	1	Left, superomedial
2005, D'Ambrosio et al. [12]	1	Left, optic strut, and anterior clinoid process
2003, Font et al. [14]	1	Bilateral, lateral, and inferior
1999, Mercado et al. [1]	1	Left, lateral and posterior, and sphenoid bone
1988, Rootman et al. [15]	1	No data, superior extending anterior cranial fossa
1985, Sebag et al. [8]	1	Right, superolateral, and roof
1984, Scully et al. [13]	1	Right, posterior, lateral, and central
1981, Hoopes et al. [9]	1	Right, superior, lateral, and posterior
1967, Sood et al. [6]	1	Left, medial, and ethmoid sinus

## Data Availability

The data that support the findings of this study are available from the corresponding author, EM-G, upon reasonable request.
